# Genome reduction of *Borrelia burgdorferi*: two TCS signaling pathways for two distinct host habitats

**DOI:** 10.1007/s11427-015-4996-z

**Published:** 2016-01-06

**Authors:** Meiping Ye, Yan Zhou, Yongliang Lou, X. Frank Yang

**Affiliations:** 1 Key Laboratory of Laboratory Medicine, School of Laboratory Medicine, Wenzhou Medical College, Wenzhou 325035, China; 2 Department of Microbiology and Immunology, Indiana University School of Medicine, Indianapolis, Indiana 46202, USA

Lyme disease is an emerging tick-borne disease in the U.S., Europe, and Asia including China, and has become the most common vector-borne disease in both Europe and North America. Infection is caused by the spirochetal pathogen *Borrelia burgdorferi* sensu lato, transmitted via tick bites. The clinical manifestations of Lyme disease range from fever and skin lesions (erythema migrans) to multisystem disorders such as arthritis, carditis, and neuroborreliosis (Steere et al., 2004). Although treatable with antibiotics, the infection is underdiagnosed, and the late stages of Lyme disease are difficult to treat. No commercial vaccine is available. An alternative strategy is to block the transmission of *B. burgdorferi*. Understanding how *B. burgdorferi* is maintained in the enzootic cycle is the key for such strategy.

*B. burgdorferi* is maintained in an enzootic cycle containing two markedly different hosts, an arthropod vector and a mammalian host (Radolf et al., 2012; Samuels, 2011). *B. burgdorferi* must be able to adapt to both distinct host environments. On the other hand, as a non-free living pathogen, *B. burgdorferi* has a dramatically reduced genome. Remarkably, *B. burgdorferi* has evolved in utilizing its limited genomic capabilities to adapt to and survive in these two host environments. Therefore, *B. burgdorferi* provides a wonderful system to study mechanism of signal transduction and host adaptation. In this regard, bacterial two-component systems (TCSs) are the main signaling pathways that bacteria utilize to sense and respond to environmental conditions. A typical TCS consists of a histidine kinase as a sensor and a corresponding response regulator that mediates the cellular response (Stock et al., 2000). Most bacteria have many TCS systems. For example, *Escherichia coli* has over 30 TCSs. In contrast, the *B. burgdorferi* genome only has two TCSs, (in addition to the chemotactic CheA-CheY system), Hk1-Rrp1 and Hk2-Rrp2. Considering that *B. burgdorferi* encounters two hosts throughout its life cycle, could *B. burgdorferi* have evolved to employ these two TCSs to survive in each of the hosts?

Uncovering the function of Hk2-Rrp2 pathway in the enzootic cycle of *B. burgdorferi* was one of the milestones in the research of molecular biology and pathogenesis of *B. burgdorferi* (Radolf et al., 2012; Samuels, 2011). In the past decade, we and others have shown that Rrp2 functions as a transcriptional regulator that activates a sigma factor cascade, the σ^54^-σ^S^ cascade. Rrp2 is a NtrC-type bacterial enhancer-binding protein (bEBP) required for activation of the alternative sigma factor σ^54^. Upon stimulation, Rrp2 becomes phosphorylated at its N-terminal response receiver domain, and phosphorylated Rrp2 and σ^54^ work together to activate transcription of *rpoS* from a -24/-12 σ^54^-type promoter. The sigma factor RpoS (σ^S^) functions as a global regulator, controlling expression of more than 15% of the genes in *B. burgdorferi*. Many of these genes encode surface lipoproteins (*B. burgdorferi* does not have lipopolysaccharide on its surface), and these lipoproteins, such as OspC, DbpA/B, and BBK32, have been shown to interact with host molecules and are required for colonization and host immune evasion (Radolf et al., 2012; Samuels, 2011).

Bodies of evidence indicate that Rrp2 is activated during spirochetal transmission from ticks to mammals. Along with other PerR/Fur-like activator BosR, phosphorylated Rrp2 activates the σ^54^-σ^S^ cascade. In addition to Rrp2 and BosR, a small RNA-binding protein DsrA, a ROC-type repressor BadR, and a plasmid-coded protein BBI16, have also been recently shown to be involved in regulation of RpoS levels in *B. burgdorferi* (Dulebohn et al., 2014; Miller et al., 2013; Radolf et al., 2012; Samuels, 2011). The Rrp2-RpoN-RpoS signaling pathway functions as a gate-keeper, is activated upon tick feeding, and controls production of many virulence factors required for the process of transmission and invasion of the mammalian host (Radolf et al., 2012; Samuels, 2011). Therefore, the Hk2-Rrp2 pathway plays a central role in *B. burgdorferi* survival in the mammalian host environment.

The function of the second TCS signaling system in *B. burgdorferi*, Hk1-Rrp1, has begun to be elucidated in the past few years. The response regulator Rrp1 is the sole diguanylatecyclase for synthesis of diguanylate (c-di- GMP). c-di-GMP is a new global second messenger found ubiquitously in the bacterial world (Römling et al., 2013). c-di-GMP is synthesized by diguanylatecyclases (DGCs), a group of GGDEF domain-containing proteins, and is broken down by phosphodiesterases (PDEs) that contain a conserved EAL or HD-GYP domain. Most free-living bacteria have numerous DGCs for synthesis of c-di-GMP, and phosphodiesterases (PDEs) for hydrolysis of c-di-GMP, and in fact, GGDEF, EAL and HD-GYP domains are among the most abundant domains encoded in bacterial genomes. Each DGC regulates its own environmental signal(s). It has been shown to play a key role in controlling the switch between the motile, single-cellular lifestyle and the sessile, multicellular lifestyle (biofilms). It is also involved in regulating virulence, antibiotic production, cell cycle regulation, and host innate immunity.

*B. burgdorferi* has a streamlined c-di-GMP signaling cascade that involves a single DGC, Rrp1, and two PDEs. Three independent groups, using either the *hk1* or *rrp1* mutant, demonstrated that, unlike the Hk2-Rrp2 pathway which is essential for mammalian infection, both the *hk1*and*rrp1* mutants were still capable of infecting the mice. However, upon acquired by ticks, neither mutant was able to survive in the tick midgut (Caimano et al., 2011; He et al., 2011; Kostick et al., 2011). Thus, the Hk1-Rrp1 pathway is dispensable for mammalian infection, but is essential for *B. burgdorferi* to survive in the tick vector.

How does Hk1-Rrp1 contribute to spirochetal adaptation to the tick host environment? It appears that one of the defects of the *hk1* or *rrp1* mutant in ticks is, in part, due to a defect in its inability to utilize glycerol, chitobiose and N-acetylglucosamine (He et al., 2011; Sze et al., 2013). Glycerol is produced by certain insects as well as arthropods as cryoprotective molecule. It was found that expression of the glycerol uptake/metabolism operon *glpFKD* is important to the fitness of spirochetes in ticks, and the *hk1* or *rrp1* mutant is defective in *glpFKD* expression (He et al., 2011; Pappas et al., 2011). Constitutive expression of *glpFKD* in the *rrp1* mutant can partially rescue the *rrp1* mutant’s survival in ticks or its transmission to the mammalian host. Chitobiose is a major component of the tick cuticle and an important source of N-acetylglucosamine for cell wall synthesis in *B. burgdorferi*. One of the chitobiose transporter gene *chbC* was shown to be defective in the *rrp1* mutant, and supplementing N-acetylglucosamine in tick midguts partially rescued the *rrp1* mutant defect in ticks (Sze et al., 2013). In addition to nutrient utilization, c-di-MGP appears to also affect the motility of the spirochetes, since the *plzA* mutant, which encodes PlzA, the only c-di-GMP binding protein identified in the *B. burgdorferi*, had abnormal swarming ability and reduced survival in ticks (Pitzer et al., 2011). More recently, Caimano et al. employed the live imaging technology and showed that the protective response mediated by c-di-GMP is multifactorial to contribute spirochetal survivals during tick feeding (Caimano et al., 2015). Further studies are warranted on how c-di-GMP modulates these diverse functions.

All evidences available thus far indicate that the two TCS systems in *B. burgdorferi*, Hk1-Rrp1 and Hk2-Rrp2, each controls spirochetal adaptation to one host environment (Figure 1). It makes biological sense that through genome reduction, only two TCSs were kept, given that *B. burgdorferi* is an obligated pathogen, not a free-living bacterium that needs to survive in many environmental conditions: ticks and mammals are the only two habitats *B. burgdorferi* has to deal with. Although each TCS is required for survival in each host, there is an interplay between the two systems. This is not surprising considering that when spirochetes are migrating from ticks to mammals during tick feeding, both Hk1-Rrp1 and Hk2-Rrp2 are activated. A common mechanism of cross-talk between TCS systems in other bacteria is that the histidine kinase sensor of one TCS phosphorylates a response regulator of the other TCS. This appears not to be the case for *B. burgdorferi*. We recent reported that the interplay between Hk1-Rrp1 and Hk2-Rrp2 is mediated through the c-di-GMP receptor PlzA (He et al., 2014). This is yet another example how *B. burgdorferi* with a streamlined genome continues unveiling its uniqueness. Further study on how the downstream targets of these two signaling systems function in each host is warranted, which will undoubtedly shed light on our understanding of vector- pathogen-host interactions.

## Figures and Tables

**Figure 1:**
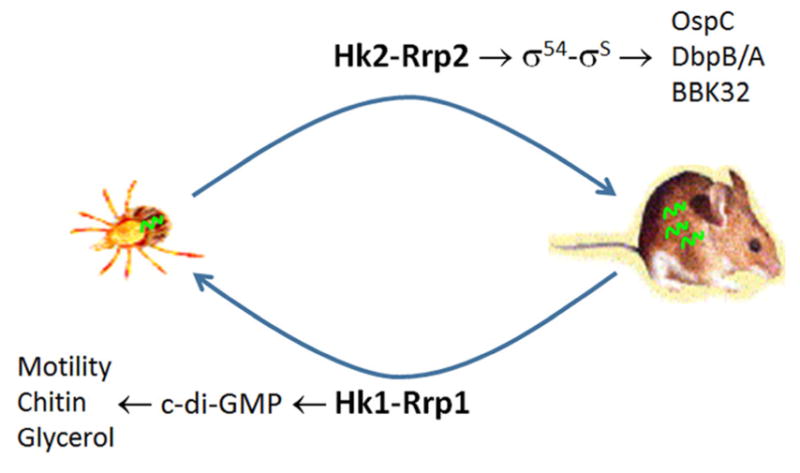
The Lyme disease pathogen *B. burgdorferi* has only two TCSs (in addition to chemotaxis systems). One system, Hk2-Rrp2, upon sensing the signals, activates genes required for mammalian infection. The other system, Hk1-Rrp1, governs multiple activities that are essential for the pathogen’s survival in the tick vector.
